# Ciliated Foregut Cyst of the Gallbladder: Report of a Case and Review of Literature

**DOI:** 10.4061/2010/193535

**Published:** 2009-12-16

**Authors:** Asiye Şafak Bulut, Kaan Karayalçın

**Affiliations:** ^1^Department of Pathology, MESA Hospital, Yasam Cad. No 5, Sogutozu, 06510 Ankara, Turkey; ^2^Department of General Surgery, Medical School of Ankara University, Sıhhiye, 06110 Ankara, Turkey

## Abstract

Cystic lesions of the gallbladder are very rare and they are generally lined by single columnar or mucinous epithelium. A ciliated cyst of foregut origin is extremely rare in gallbladder. To our knowledge, only five cases have been reported so far. Here, we present the sixth case found incidentally in ultrasonographic examination in a 41-year-old woman suffering from chronic right upper quadrant pain. Laparoscopic cholecystectomy was performed for the gallstones and a benign appearing cyst in ultrasonography. Macroscopically, a submucosal unilocular cyst was located in the neck of the gallbladder. There was no communication with the lumen. Histologically, the cyst was lined by pseudostratified ciliated epithelium containing goblet cells and had a muscular wall. The postoperative course was uneventful. Patient was discharged on the second day of the operation and was well after 2 months.

## 1. Introduction

Ciliated foregut cyst is an uncommon developmental anomaly that usually develops above the diaphragm in the form of bronchial and esophageal cysts. Foregut cysts below the diaphragm are very rare and generally found in the liver [1]. Extrahepatic cases are much rarer. To our knowledge, only five cases of ciliated foregut cyst of the gallbladder were reported so far [2–6]. Here we report the sixth case in literature.

## 2. Materials and Methods

Cholecystectomy material was fixed in 10% formaldehyde. Representative slices were obtained and embedded in paraffin. The paraffin blocks were cut into 5 m thick sections and stained with hematoxylin and eosin. Periodic acid-Schiff stain was used for histochemistry. Smooth muscle actin (SMA), estrogen receptor (ER), and progesterone receptor (PR) antibodies were used for immunohistochemistry.

## 3. Results

### 3.1. Case Report

A 41-year-old woman was admitted to our hospital with right upper quadrant pain. Laboratory data of the liver function tests, biliary enzymes, and serum tumor markers were normal. Abdominal ultrasonography revealed gallstones and a benign appearing cystic lesion located in the neck of the gallbladder. Laparoscopic cholecystectomy was performed. 

**Figure 1:**
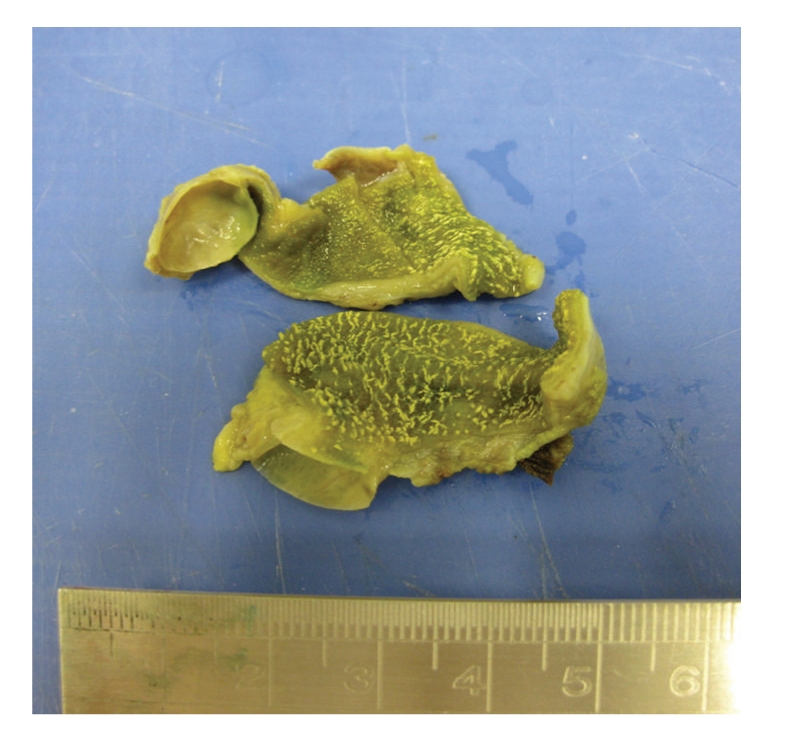
Gross appearance of a unilocular intramural cyst located under the mucosa showing diffuse cholesterosis.

Macroscopic examination of the specimen revealed a submucosal unilocular cyst, located in the neck of the gallbladder, measuring 3.5 × 2 × 1.5 cm, without communication to the lumen (Figure 1). The cyst contained blurry mucoid fluid. Its wall was thin and the inner surface was smooth. Gallbladder mucosa showed diffuse linear yellow streaks compatible with cholesterosis. 

**Figure 2:**
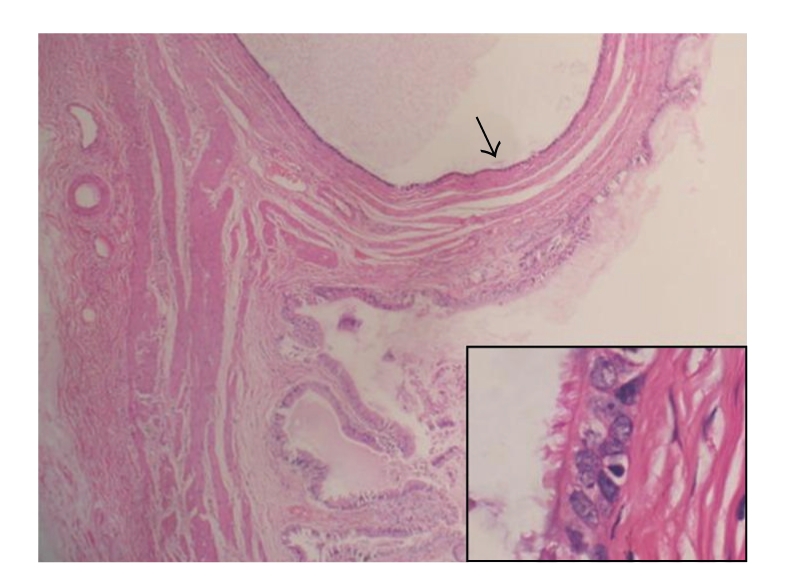
The cyst is lined by a pseudostratified ciliated columnar epithelium and has an underlying smooth muscle wall (HE, Original magnification is ×40, inset ×1000).

**Figure 3:**
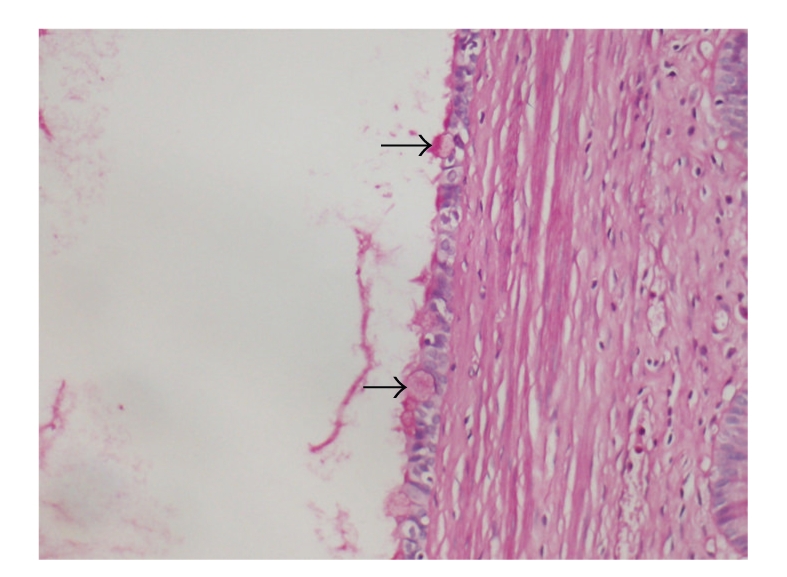
The cyst epithelium containing a few goblet cells (arrows) (Periodic asid-Schiff, Original magnification is ×200).

**Figure 4:**
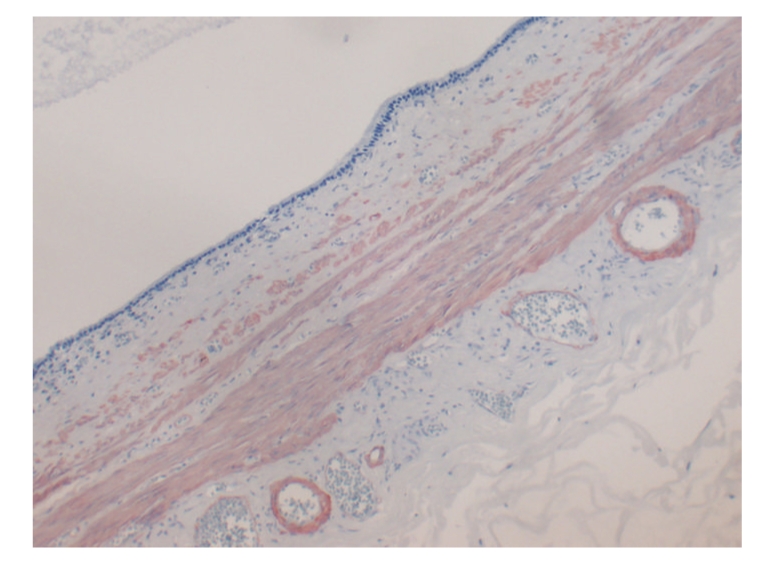
SMA expression in the cyst wall with its positive control in the vascular smooth muscle (Immunohistochemistry, Original magnification is ×100).

In the microscopic examination, the cyst wall was made up of muscular tissue and it was lined by pseudostratified ciliated epithelium (Figure 2). The epithelium contained a few goblet cells staining bright magenta by periodic acid-Schiff stain (Figure 3). Neither ER nor PR expression was observed in the epithelium. The smooth muscle wall stained with SMA immunohistochemically (Figure 4). Metaplastic or dysplastic changes were not observed in the epithelial cells. Collections of lipid-filled foamy cells were present in the tips of the villi. There was chronic inflammation within the lamina propria. A diagnosis of ciliated foregut cyst and chronic calcareous cholecystitis with cholesterosis was made. 

The postoperative course was uneventful. The patient was discharged on the second date of the operation and was well after 2 months.

## 4. Discussion

Cystic lesions of the gallbladder are rare. They include acquired, neoplastic, or congenital cysts. Ciliated foregut cysts are congenital cysts arising from the remnants of the embryonic foregut and are extremely rare in the gallbladder. To our knowledge, only five cases were reported so far [2–6]. Kakitsubata et al. firstly described a gallbladder cyst lined by a single layer of ciliated columnar epithelium with a fibro-muscular wall and used the term “epithelial cyst of the gallbladder” in 1995. They suggested an origin of Luschka's duct [2]. Benlolo et al. presented 4 patients with ciliated hepatic cysts, one of which was found in the gallbladder wall in 1996 [3], but they did not attribute a distinct name for the one in the gallbladder. In 2000, Nam et al. presented a ciliated cyst located in the gallbladder and suggested the term “ciliated foregut cyst of the gallbladder” firstly [4]. Hirono et al. [5] and Muraoka et al. [6] then reported 2 additional cases in 2002 and 2003. As far as we know, this is the sixth case in literature. In the previously reported cases, patients were mostly women and cysts were located in the fundus and cervix of the gallbladder. All of them were unilocular cysts containing mucinous or thick fluids. Their size ranged between 1.1 and 3 cm. They had smooth muscles in their wall and lined by a ciliated pseudostratified epithelium. They were located under the mucosa and did not communicate with the lumen. Our case's clinical and histological features are all compatible with the reported ones. 

Although no serous cysts or endosalpingios had been reported so far in the gallbladder, these are considered in the differential diagnosis for their epithelial resemblance to our case, as most of the reported ciliated foregut cysts are from female patients. But unlike these lesions, our case has its own smooth muscle wall and the epithelium lack ER or PR positivity immunohistochemically.

Among the subdiaphragmatic organs, ciliated foregut cysts are mostly located in the liver (1). They are generally asymptomatic benign lesions and they generally do not communicate with the gallbladder although there is only one case communicating with the gallbladder [7]. Squamous metaplasia and even squamous cell carcinoma may arise in a hepatic foregut cyst [8, 9]. Laparoscopic excision is preferred for the treatment, as a minimally invasive procedure [10].

The presented case is a rare case of ciliated foregut cyst located in the gallbladder. Such a cyst must be considered in the differential diagnosis of a gallbladder cyst. Although malignant transformation was not reported so far, squamous cell carcinoma must be considered during the microscopic examination as it was reported in a ciliated hepatic foregut cyst [9].
